# Optimization of Integrated Impeller Mixer via Radiotracer Experiments

**DOI:** 10.1155/2014/242658

**Published:** 2014-03-06

**Authors:** N. Othman, S. K. Kamarudin, M. S. Takriff, M. I. Rosli, E. M. F. Engku Chik, M. A. K. Adnan

**Affiliations:** ^1^Department of Chemical and Process Engineering, Faculty of Engineering and Built Environment, Universiti Kebangsaan Malaysia, 43600 Bangi, Selangor Darul Ehsan, Malaysia; ^2^Malaysian Nuclear Agency, 43000 Kajang, Selangor Darul Ehsan, Malaysia

## Abstract

Radiotracer experiments are carried out in order to determine the mean residence time (MRT) as well as percentage of dead zone, *V*_dead_ (%), in an integrated mixer consisting of Rushton and pitched blade turbine (PBT). Conventionally, optimization was performed by varying one parameter and others were held constant (OFAT) which lead to enormous number of experiments. Thus, in this study, a 4-factor 3-level Taguchi L9 orthogonal array was introduced to obtain an accurate optimization of mixing efficiency with minimal number of experiments. This paper describes the optimal conditions of four process parameters, namely, impeller speed, impeller clearance, type of impeller, and sampling time, in obtaining MRT and *V*_dead_ (%) using radiotracer experiments. The optimum conditions for the experiments were 100 rpm impeller speed, 50 mm impeller clearance, Type A mixer, and 900 s sampling time to reach optimization.

## 1. Introduction

Radiotracer technology has enabled engineers and technologists to troubleshoot malfunctions, mechanical damage, and process anomalies without plant shutdown or disruption to operating processes and has received broad acceptance worldwide. The application of radioisotope-based technology is rapidly growing role in assisting industry in enhancing their production efficiency and also in plant operation optimization. This technology makes it possible to satisfy the critical need for production efficiency through the investigation of process problems. Nevertheless, the application of radiotracer technology among Malaysia industries is minimal. The industries are reluctant to use the radiotracer technology due to safety concerns. Bromine-82, which emits gamma energy with a half-life of 36 hours and 0.55 (70%) and 1.32 MeV (27%), is the most commonly used radiotracer in the oil and gas industries.

Apparently, it is undeniable that radiotracer technique is the most reliable tool to detect plant anomalies and plant optimization without plant shut down [1–3]. Nevertheless, the repeated radiotracer experiments for optimization of a unit process will cause unnecessary exposure of radiation emission to the operators. Moreover, in the past, the numbers of runs were carried out based upon tracer engineers' experiences. Thus, a L9 orthogonal array of Taguchi method is introduced in this study to ensure minimal radiation exposure with accurate optimization assurance with least number of trials.

Many has used the Taguchi method which is a statistical approach for the purpose of designing and improving product quality of industrial plants by implementation of orthogonal arrays, in order to reduce number of experiments due to cost and time constraints such as in selection of process parameters in strontium ferrite sintered magnets [4], determination of bromine chemistry etching recipe for deep silicon trenches [5], optimization of cutting parameters of glass fibre reinforced plastic (GFRP) composite [6], evaluation of granule processes [7], optimization of thin-film sputtering process in color filter manufacturing [8], and optimization of plasma surface hardening [9]. The rapid progress of Taguchi orthogonal array in research and manufacturing for multiapplications has facilitated the results of optimization to be obtained faster with minimal costs. This is because the traditional method of optimization will require one parameter to be varied while keeping the rest constant (OFAT). The action with conventional parametric design of experiment approach is time consuming and calls for enormous resources especially when the numbers of parameters are increased [4]. According to Khoei et al. [10], the key concept of the Taguchi method is to optimize process parameters and to achieve high quality of products with low cost because it can reduce research and development costs by simultaneously studying a large number of parameters [10]. As agreed by Kamil et al. [11] who conducted optimization of polyol ester production, the advantage of using Taguchi design is that the method allows several effects of factors to be simultaneously determined effectively and efficiently. Besides, the optimization of the process parameters by Taguchi method implemented the analysis of signal-to-noise (SN) ratio table and graph to identify the impact rank of the listed factors as well as to investigate the simultaneous variation of several parameters and the investigation of interactions between parameters [5, 9].

## 2. Experimental Procedure 

**Figure 1:**
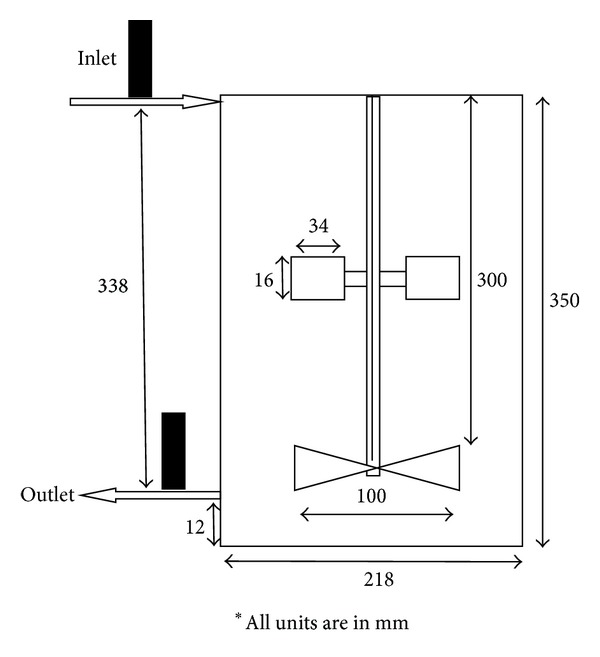
Schematic diagram of Type A mixing vessel rig for radiotracer experimentation.

**Figure 2:**
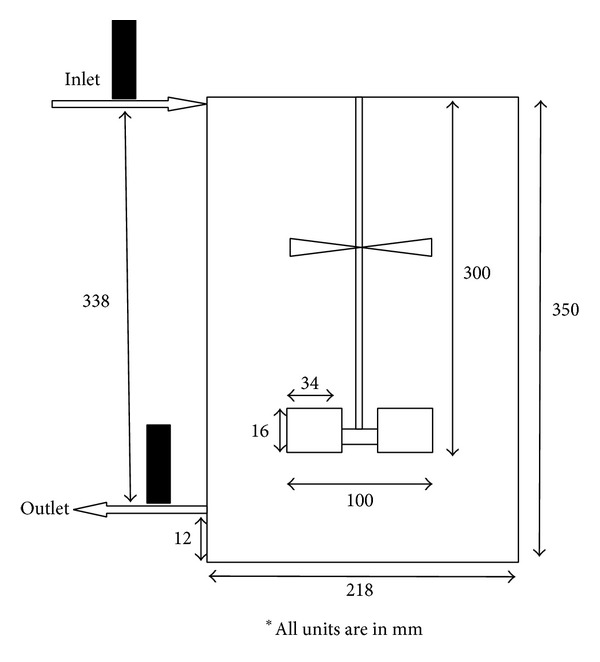
Schematic diagram of Type B mixer.

In this study, Taguchi orthogonal array design, a robust design method, was developed for reducing cost and improving mixing efficiency in mixed axial and radial impeller of mixing vessel safely and accurately. Two types of integrated continuous flow rig were used throughout this study and denoted as Type A and Type B. A total of nine runs were carried out prior data analysis by varying the impeller clearance, the speed of impeller, and the sampling time, and each experiment was conducted in random order to reduce statistical error. The parameters studied were variations of impeller clearance from 50, 75, and 100 mm, impeller speed which were 100, 150 and 200 rpm, and sampling time from 700, 800 and 900 s, respectively, with respect to percentage of dead zone and MRT. The results were then treated using MATLAB R2010b to eliminate the parasitic source. The experiments were conducted with two different types of impellers attached to a common shaft: a Rushton turbine and a pitched blade turbine (PBT) for the radial and axial impeller, respectively. In this study, the Type A mixer was assigned to the Rushton turbine that was mounted above the PBT whereas for Type B was assigned to PBT which was mounted above the Rushton turbine. Two types of impeller and three control factors with three levels were selected. Based on the Taguchi quality design concept, an L9 orthogonal array table was chosen for this research as shown in Table 1. The Taguchi method was applied initially to plan an optimum number of experiments for process optimization since the radioactive experiments were involved. The sodium iodide detectors were positioned at the inlet and outlet and connected to a data acquisition system (DAS). The DAS monitored the movement of the injected Tc-99m in the mixer during operation. Approximately, 10 *μ*Ci of Tc-99m was used for each run. The Tc-99m was diluted to ensure that the radiation exposure was minimal to reduce the radiation risk to the operator. The schematic of both rigs is shown in Figures 1 and 2.

## 3. Results and Discussion

**Figure 3:**
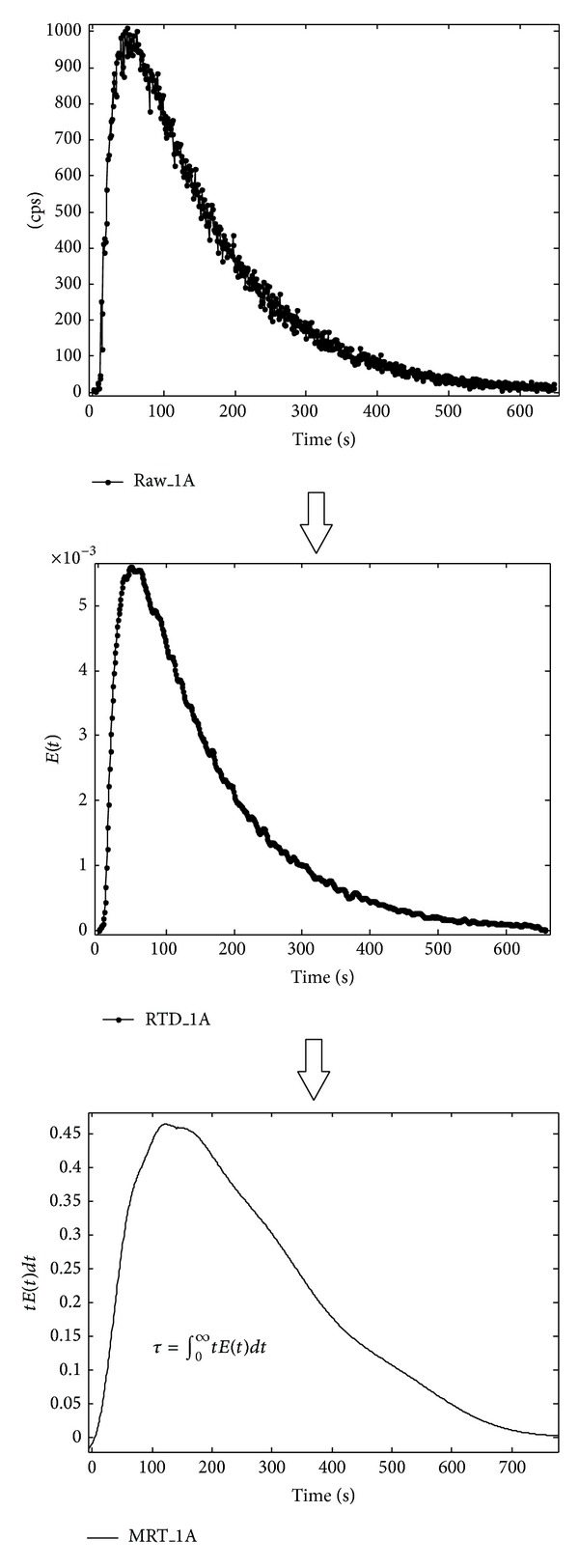
Determination of mean residence time (MRT).

By choosing L9 orthogonal array, the interactions between factors are not considered in view to cost and time saving (4, 5). The results obtained from radiotracer experiments were in the form of counts per second (cps) versus time. The curves obtained were then normalized into RTD curves before translating them to MRT curves. The calculation of MRT was made by integrating the area under the curves of the respective graphs as shown in Figure 3. The pretreatment of data was made prior to plotting the RTD curves as described by Kasban et al. [12].

The application of L9 (3^4^) Taguchi orthogonal array has produced nine number of experimental runs as shown in Table 2. Each run was conducted randomly to reduce potential statistical error. The summarized results of each run were shown in Table 3. According to this table, experiment number 4 which was Run 7A produced highest value of MRT with minimum percentage of dead zone. Thus, Taguchi method will be carried out to verify the results. In Taguchi method, the signal to ratio (*S/N*) should be calculated to determine the optimal condition of the respective experiments. Nevertheless, due to safety concerns, the replication of radiotracer was not conducted in order to avoid unnecessary radiation exposure to the operators. Thus, means of each level for four factors will be calculated and the assessment is still acceptable as done by Chen et al. [5]. Besides the uncertainty in using radiotracer, the accuracy of the experimental results has been proven to be around 1-2% [13].

### 3.1. Effect from Four Factors

Taguchi design recommends analyzing the mean response for each run in the inner array. Besides, the means of four factors as substitute of *S/N* will determine which factors give the greatest influence in optimization.  Table 4 shows the means of each level for four factors. It can be seen from Table 4 that the highest effects among the four factors are type of mixer at Level 1, impeller speed at Level 1, impeller clearance at Level 1, and sampling time at Level 3. In this study, in order to obtain optimal condition for each parameter, the MRT has to be maximum, whereas *V*_dead_ has to be minimum in value. Thus, the optimum condition was reached with A_1_B_1_C_1_D_3_ for both MRT and *V*_dead_ (%) where the optimum settings for the experiments were 100 rpm impeller speed, 50 mm impeller clearance, Type A mixer, and 900 s of sampling time which is considered as sufficient time for collected signals to reach background readings. The selection of hybrid impeller by Sahle-Demessie et al. 2003 has reported that the use of axial or mixed impellers can improve the flow profile by narrowing the RTD curves, avoiding back mixing and creating a high Reynolds number [14]. To date, no studies have been conducted for dual integrated impeller mixer using Rushton and PBT. Moreover, according to Vrábel et al. (2000), the combination of axial/radial impeller (i.e., PBT/Rushton) in a mixer will lead to great improvement of axial mixing compare to using Rushton turbines only [15]. Therefore in this study, it showed that Type A mixer contributed to better optimization condition.

### 3.2. Effects of Impeller Speed on RTD

**Figure 4:**
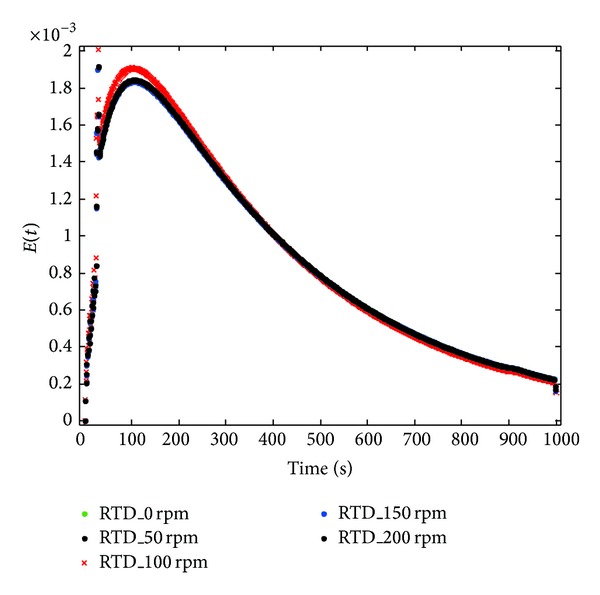
Effects of impeller speed on RTD.

For verification purpose, a short simulation using computational fluid dynamics (CFD) was carried out to study the effect of speed on the residence time distribution (RTD). The speed of impeller was varied from 50, 100, 150, and 200 rpm, respectively. Very minimal change can be observed as shown in Figure 4. Nevertheless, the impeller speed of 100 rpm showed the highest peak compared to others. Thus, 100 rpm was chosen as optimum parameter for process optimization. Moreover, the Rushton turbine is able to produce two symmetrical loops on the top and bottom of the impeller, whereas the PBT can produce a pair of circulation loops on its side. Perhaps, the presence of both radial Rushton turbines and the axial turbine (PBT) has improved the fluid circulation during mixing.

Critical impeller arrangements can lead to parallel, merging, or diverging flow profiles. The flow configurations can be described by defining the lower impeller clearance *C*, impeller spacing *S*, and both are normalized with tank diameter *T*. In this study, the value of *S*/*T* is 0.7 and *C*/*T* is 0.24. According to Montante and Magelli (2004), this ratio indicates that the flow pattern produced by single impeller will not interrupt with adjacent impeller. Thus, the flow profile produced by this arrangement is parallel flow [16]. Parallel flow will ensure the flow pattern by each impeller is similar with a single impeller. Moreover, homogenization in mixing operation is best practice when the flow field formed from each impeller is wide enoughand no interaction between adjacent impellers. Hence, 50 mm was chosen as the best selection of optimum parameter for mixing optimization. The 900 s was selected as optimum time compared to others since sufficient time was needed for radioactive counts to return to its background reading so that the contaminated fluid in the vessel during experimentation was safe to be discharged upon completion.

### 3.3. ANOVA

In order to study the significance of the factors on the observed value, the statistical analysis of the data was performed by analysis of variance (ANOVA). ANOVA enables one to study the contribution of the factor and interactions as well as to explore the effects of each process on the observed value besides to determine the relative importance of various factors [10]. The results obtained from the ANOVA were tabulated in Table 5. It can be seen clearly from Table 5 that type of mixer produced significant contribution towards the optimization of MRT (70.19%) as well as *V*_dead_ (71.68%). Moreover, values of Prob > *F* less than 0.05 indicate that all factors involved are significant. The *R* ^2^ of 0.95 for both outputs are obtained implying that 95% of factors can be fitted well into the model.

### 3.4. Experiments at Optimum Condition

In order to verify that the optimum conditions suggested by the matrix experiments do indeed give the projected improvement, hence, an experiment was conducted using the predicted optimum levels for the control parameters being studied. If the observed and the projected improvements match, the suggested optimum conditions will be adopted. The optimum parameter setting used in the validation experiments and the results are presented in Table 6. According to ANOVA, type of mixer has the highest significant effect to the MRT and *V*_dead_; thus, additional experiment was carried out for Type B mixer with similar optimization setting for comparison purpose. Nevertheless, the results have shown that Type A mixer with optimum condition has enabled to increase the value of MRT as well as reducing the percentage of dead zone in the mixing operation. Besides, the obtained results also show that Taguchi method and the use of integrated mixer produced better results than other technique as shown in Table 7. According to the table, the use of jet mixing produced experimental MRT greater than theoretical MRT. This situation indicates that the tracer is not representing the fluid in the system or under estimated the calculation of theoretical MRT. Besides, study by Choi et al. [17] showed that by using single Rushton in 1.3l unbaffled vessel, the optimum percentage of dead volume achieved was 7% with respective parameters, whereas no specific values of important parameters have been stated for MRT estimation by IAEA [13]; Cao et al. [18]; and Furman et al. [19].

## 4. Conclusion

In this study, a 4-factor 3-level Taguchi L9 orthogonal array was introduced to obtain accurate optimization with minimal number of experiments. The optimal condition of four process parameters, namely, the type of mixer, speed, impeller clearance, and sampling time, were assessed based on MRT and *V*_dead_ (%) using radiotracer experiments. The optimum settings for the experiments were 100 rpm impeller speed, 50 mm impeller clearance, Type A mixer, and 900 s sampling time which indicates sufficient length of signal collection to reach optimization. Moreover, ANOVA was performed to determine the most influential factors contributed in this study. Values of Prob > *F* less than 0.05 indicate that all factors involved are significant. The *R* ^2^ value (0.95) for both outputs implies that 95% of factors can be described well by the model. The predicted optimum levels for the control parameters for Type A mixer as well as additional run for Type B mixer were validated by conducting an experiment at these conditions. In conclusion, Taguchi L9 orthogonal array has been successfully carried out in assisting optimization in radiotracer experiments.

## Figures and Tables

**Table 1:** The value of level of selected parameters in this study.

Factors	F1: type mixer	F2: impeller speed (rpm)	F3: impeller clearance (mm)	F4: sampling time (s)
Level 1	A	100	50	700
Level 2	B	150	75	800
Level 3	—	200	100	900

**Table 2:** The application of L9 (3^4^) Taguchi orthogonal array has produced nine numbers of experimental runs.

Experiments	F1: type mixer	F2: impeller speed (mm)	F3: impeller clearance (rpm)	F4: sampling time (s)
1	Level 1	Level 1	Level 1	Level 1
2	Level 1	Level 2	Level 2	Level 2
3	Level 1	Level 3	Level 3	Level 3
4	Level 2	Level 1	Level 2	Level 3
5	Level 2	Level 2	Level 3	Level 1
6	Level 2	Level 3	Level 1	Level 2
7	Level 3	Level 1	Level 3	Level 2
8	Level 3	Level 2	Level 1	Level 3
9	Level 3	Level 3	Level 2	Level 1

**Table 3:** The summarized results of MRT and *V*_dead_.

Experiments	Number of runs	MRT (s)	*V*_dead_ (%)
1	1A	154.25	1.58
2	3A	136.40	12.97
3	5A	151.54	3.31
4	7A	156.17	0.35
5	8A	141.31	9.83
6	2B	128.78	18.60
7	4B	129.85	18.14
8	6B	131.38	17.0
9	9B	134.57	14.30

**Table 4:** The means of each level for four factors.

Parameters	Factors	A: type mixer	B: impeller speed (mm)	C: impeller clearance (rpm)	D: sampling time (s)
MRT (s)	Level 1	147.40	146.76	142.38	143.38
	Level 2	142.09	136.36	138.14	131.68
	Level 3	131.93	138.30	140.9	146.36

*V*_dead_ (%)	Level 1	5.95	6.69	9.21	8.57
	Level 2	9.59	13.27	12.39	16.57
	Level 3	16.48	12.07	10.43	6.89

**Table 5:** ANOVA results for radiotracer experiments.

Factors	SS	d.o.f.	Mean square	%contribution	Prob > *F*
(1) MRT (s)					
Type mixer	661.95	1	661.95	70.19	0.0069
Speed	197.89	2	98.95	20.98	0.0476
Clearance	38.32	2	19.16	4.06	0.0314
Time	29.86	2	14.93	1.60	0.0338
Std. deviation	3.87				
*R* ^2^-squared	0.9524				
Mean	140.47				

(2) *V*_dead_ (%)					
Type mixer	301.95	1	301.95	71.68	0.0087
Speed (rpm)	79.16	2	39.58	18.79	0.0440
Clearance	20.34	2	10.17	4.83	0.0337
Time	131.39	2	6.59	3.13	0.0419
Std. deviation	2.76				
*R* ^2^-squared	0.9448				
Mean	10.53				

**Table 6:** Parameter setting and results obtained from confirmation experiment.

Type mixer	Clearance (mm)	Speed (rpm)	Sampling time (s)	MRT (s)	*V*_dead_ (%)
A-L1	50-L1	100-L1	900-L3	156.05	0.39
B	50	100	900	139.44	11.87

**Table 7:** Comparisons of current technique using Taguchi method and other method.

Authors	Method	Parameters					Results		
		*S* (rpm)	*C* (mm)	*H* (mm)	*D* (mm)	*Q* (lpm)	MRT (theo)	MRT (exp)	*V*_dead_ (%)
Current study	Integrated mixer (dual impeller)	100	50	350	21.8	5	156.72	156.05	0.39
[17]	Single Rushton	288	—	145	114	0.53	2.64	2.445	7.4
[12]	Four stirrer paddles	—	—	800	300	525	—	3420	—
[18]	Multibladed T-type blade	50	—	2 m	0.186 m	30	—	426.36	—
[19]	Jet mixing		—	—	—	1.92	356	363	!

*S*: speed, *C*: clearance, *H*: height, *D*: diameter, *Q*: volumetric flow rates, MRT: mean residence time, theo: theoretical, and exp: experimental.
